# Non-Medical Risk Factors as Avoidable Determinants of Excess Mortality in Children with Chronic Kidney Disease. A Prospective Cohort Study in Nicaragua, a Model Low Income Country

**DOI:** 10.1371/journal.pone.0153963

**Published:** 2016-05-12

**Authors:** Giovanni Montini, Alberto Edefonti, Yajaira Silva Galán, Mabel Sandoval Díaz, Marta Medina Manzanarez, Giuseppina Marra, Fabio Robusto, Gianni Tognoni, Fabio Sereni

**Affiliations:** 1 Pediatric Nephrology and Dialysis Unit, Fondazione IRCCS Ca' Granda Ospedale Maggiore Policlinico, Milano, Italy; 2 Pediatric Nephrology and Dialysis Unit, Hospital Infantil Manuel de Jesus Rivera “La Mascota”, Managua, Nicaragua; 3 Fondazione Mario Negri Sud, Santa Maria Imbaro, Italy; 4 Department of Clinical Sciences and Community Health, University of Milan, Milan, Italy; The University of Manchester, UNITED KINGDOM

## Abstract

**Background:**

The widely recognized clinical and epidemiological relevance of the socioeconomic determinants of health-disease conditions is expected to be specifically critical in terms of chronic diseases in fragile populations in low-income countries. However, in the literature, there is a substantial gap between the attention directed towards the medical components of these problems and the actual adoption of strategies aimed at providing solutions for the associated socioeconomic determinants, especially in pediatric populations. We report a prospective outcome study on the independent contribution and reciprocal interaction of the medical and socioeconomic factors to the hard end-point of mortality in a cohort of children with chronic kidney disease in Nicaragua.

**Methods and Findings:**

Every child (n = 309) diagnosed with chronic kidney disease (CKD) and referred to the tertiary unit of Pediatric Nephrology in Managua (Nicaragua) from a network of nine hospitals serving 80% of the country’s pediatric population was registered between January 2005 and December 2013. The three main socioeconomic determinants evaluated were family income, living conditions and the family’s level of education. Further potential determinants of the outcomes included duration of exposure to disease, CKD stage at the first visit as suggested by the KDOQI guidelines in children, the time it took the patients to reach the reference centre and rural or urban context of life. Well-defined and systematically collected medical and socioeconomic data were available for 257 children over a mean follow-up period of 2.5±2.5 years. Mortality and lost to follow-up were considered as outcome end-points both independently and in combination, because of the inevitably progressive nature of the disease. A high proportion (55%) of children presented in the advanced stages of CKD (CKD stage IV and V) at the first visit. At the end of follow-up, 145 (57%) of the 257 cohort children were alive, 47 (18%) were lost to follow-up and 65 (25%) had died. Cox regression analysis showed an independent contribution to mortality of CKD stage at diagnosis and of level of education, with overlapping HR values (HR and 95%CI: 2.66; 1.93–3.66 and 2.72; 1.71–4.33, respectively).

**Conclusions:**

The unfavourable socioeconomic and cultural background of the pediatric study cohort and the severity of kidney damage at diagnosis were the key determinants of the clinical risk conditions at baseline and of the mortality outcome. Long-term structural interventions on such backgrounds must be adopted to assure effectiveness of medical care and to assure an earlier diagnosis of CKD in these patients. The translation-extension of our results is currently underway with an agenda which includes: 1) better integration of chronic pediatric conditions into primary care strategies to promote prevention and early timely referral; 2) the consideration of socioeconomic conditions as a mandatory component of the packages of best-care; 3) the formulation and flexible adaptation of guidelines and educational programs, based on the information generated by a context-specific, epidemiological monitoring of needs and outcomes, guaranteed by an effective database.

## Introduction

Over the last several years, the importance and significance of the social and economic determinants of health-disease conditions have received renewed attention in the international literature, as well as in reports by international health and human rights agencies[1]. The main focus has been concentrated on the specific relevance of these determinants in relation to non-transmissible chronic diseases (NTCDs) in low-income countries (LICs), where healthcare is often compromised by the combined effects of non-accessibility to and non-compliance with long-term medical interventions[2–4].

While the regular and detailed updates on the global burden of disease provide the information necessary for forecasting and recommending general policies, very few country-, disease- and age- targeted studies documenting the concrete interaction between medical and sociocultural conditions in the determination of health outcomes[5–8] are available. Despite their severity, pediatric NTCDs are specifically at risk of remaining orphan conditions because their relative rarity does not attract sufficient economic and human investments in research and care, specifically in LICs.

We report the results of a prospective cohort of children with chronic kidney disease (CKD) from Nicaragua, a typical LIC, whose specific needs could not find a place even in the most recent and comprehensive reviews in view of the re-formulation of the post-2015 agenda[9, 10].

## Populations and Methods

### Setting

Nicaragua is a small LIC in Central America with a census population of 6.1 million as of 2013; 51% of the population is under 19 years of age and the per capita income is among the lowest in the world[11]. The National Health Service was established in the early ‘80s and delivers countrywide medical care and assistance although it has never been supported by the systematic investments necessary for its implementation and growth. Access to care thus shares the major limitations typical of economically marginal countries: pediatric patients with clinical conditions, such as CKD, are at risk of becoming therapeutic orphans.

In 2000, the area of pediatric nephrology and urology was a model example of this, with only 3 pediatric nephrologists working in the country and practically no competences or resources available (from renal biopsies to any strategy for renal replacement). A further obstacle was the total absence of an epidemiological profile on the prevalence of the problem and, even more so, on its territorial dispersion and the socioeconomic conditions of the target populations.

The plan proposed by the group based in Milan and accepted by the Government included an agreement to ensure free access to nephrological care as an explicit medium-term goal starting, however, with a top-down approach—the establishment of a reference centre for Pediatric Nephrology and Urology, with a central core group of trained personnel at the children’s hospital in Managua (Hospital Infantil Manuel Jesus de Rivera)[12, 13]. Thirteen doctors and nurses were trained mainly in Italy and also in Costa Rica, thus permitting the progressive extension of the program into a nationwide network involving nine of the district hospitals. The network serves almost 80% of the country’s pediatric population and refers patients to the central hospital in Managua, where the essential packages of medical care were made progressively available as early as 2002. Specifically a chronic peritoneal dialysis program was initiated in 2002, followed by hemodialysis facilities in 2005. The first living related donor kidney transplant was performed also in 2005 and to date, 37 successful transplant have been carried out. A cadaveric donor transplant program is in the pipeline. It has only recently been possible to activate a parallel program of community-based activities aimed at developing a broader, prevention-focused training of the healthcare workers and the general population.

The Italian team guaranteed a minimum of 2 working weeks a year for the supervision of the project activities over the whole study period. An online database connecting Managua to the nine district hospitals was prospectively implemented for the storage of demographic information as well as clinical and laboratory data. Reliable data collection of the socioeconomic profiles of the children and their families began in 2005, thanks to the ad hoc training of the dedicated social services established at the beginning of the program.

### Patients and Social Factors

Patients were all children diagnosed with chronic kidney disease and referred to the tertiary unit in Managua between 2005 and 2013. The three main socioeconomic determinants, evaluated by a social worker who was part of the nephrology team, were family income, living conditions and parents’ level of education. The data were not collected specifically for the purposes of this research study, but were collected primarily for determining those families in need of social and economic assistance. On the basis of the existing national recommendations[11, 14, 15] the socioeconomic variables were defined and coded into three levels (acceptable, low, very low) as follows:

1. Family income of ≥ 400 US dollars/month was considered as acceptable; 141–399 USD as low; ≤ 140 USD as very low.
2. The scoring of living conditions, as described in detail in Table 1, provided the reference scheme for classifying as acceptable houses possessing at least 4 of the criteria from group 1, as very low if they had at least 4 criteria from group 3, all other combinations were coded as low.
3. The family’s level of education was classified as very low if parents were illiterate, as low for elementary school education and as acceptable for any higher educational level.

**Table 1:** Living conditions scoring system.

	Basic housing scoring criteria					
	Roofing	Walls	Flooring	Sewage system	Water supply	Power supply
**Group 1**	zinc	concrete	ceramic or brick	WC with sewage system or septic tank	potable from aqueduct	electricity
**Group 2**	tiles	wood and concrete mix	tiles or wood	latrine, cesspit	well	electricity
**Group 3**	plastic, palm leaves, nicalit*	wood, zinc, plastic	bare earth	open air	river, stream	no electricity

*Nicalit: cement and asbestos fibres

Further potential determinants of the outcomes included duration of exposure to disease (years: <1, 1–3, 4–6, 7–8), CKD stage at the first visit as suggested by the KDOQI guidelines in children[16], the time it took for patients to reach the reference centre: (< 2h, 2–6 h and >6 h); rural or urban context of life.

### Outcome measurements

The proportion of patients retained in the program and of those lost to follow-up were considered as stratification criteria and prognostic indicators of mortality. Mortality and lost to follow-up were considered as outcome end-points both independently and in combination, because of the inevitably progressive nature of the disease and the necessity for life-saving procedures in the advanced stages. This is particularly true in this context as 64% of children lost to follow-up were in KDOQI stages 4 and 5 at entry.

#### Statistical analysis

Patients’ baseline characteristics were reported as frequency (percentage) and mean±standard deviation (SD). Follow-up time was defined as the time between the diagnosis of CKD and the last contact with the individual patient. A logistic regression model based on socio-demographic data was constructed to evaluate mortality risk in the whole population. In patients with complete follow-up information, time-to-death analyses were performed using multivariate Cox proportional hazard regression models, and risks were reported as hazard ratios (HRs) along with their 95% confidence interval (CI). Survival curves and probabilities were reported according to the Kaplan-Meier method. All statistical analyses were performed using SAS Software Release 9.3 (SAS Institute, Cary, NC).

### Ethics

Data were obtained from the database in an anonymized form; anonymization was performed by the local researchers. The research Ethics Committee at the Fondazione Policlinico in Milan and the National Health Services authorities in Nicaragua determined that this study was exempt from the requirement for approval.

## Results

309 CKD children were diagnosed and registered in the database between January 2005 and December 2013. The study cohort was restricted to 257 subjects, as well defined and systematically collected socioeconomic data were not available for 52 of the children, even though their demographic and clinical characteristics were very similar to those of the study cohort. In particular, the mean ages were 10.1±5.3 and 9.6±05.5 years and the percentage of girls was 42% and 44% in the full cohort and in the studied cohort, respectively. As 19% and 18% of children were in stage IV, and 36% and 37% were in stage V in the full cohort and in the studied cohort, respectively, disease severity was also similar in both groups. The underlying causes of CKD in the study cohort were as follows: glomerulopathies 70 (27%), 58 (22%) of which were steroid resistant nephrotic syndrome; hypodysplasia with associated uropathies 26 (10%) and with no associated uropathies 26 (10%); neurogenic bladder 32 (13%); miscellaneous 19 (7%) and 84 (33%) of unknown origin. Major comorbidities, such as neurologic and cardiologic anomalies or genetic syndromes were present in only 13 (5%) cases.

At the end of the follow-up period (mean follow-up: 2.5±2.5 years), 145 (57%) of the 257 cohort children were still alive (10 of whom had been transferred to adult units due to their age), 47 (18%) were lost to follow-up and 65 (25%) had died. We can assume that the cause of death for these children was ESRD related, because of the progressive nature of the disease.

The distribution of the cohort population according to its socioeconomic descriptors is represented in Table 2, while Table 3 provides a comprehensive and detailed profile of the cohort according to the main demographic, clinical, socioeconomic factors, and according to their outcomes.

**Table 2:** The social profile of the cohort of 257 CKD paediatric patients.

	No.	%
**Living conditions score**		
Very low	33	12.8
Low	131	51.0
Acceptable	93	36.2
**Parents' education**		
Very low	41	15.9
Low	129	50.2
Acceptable	87	33.9
**Income**		
Very low	172	66.9
Low	61	23.7
Acceptable	24	9.3
**Time to reach the reference centre**		
< 2h	109	42.4
2–6 h	124	48.3
>6 h	24	9.3
**Context of life**		
Rural	101	39.3
Urban	156	60.7

**Table 3:** Profile of the cohort according to its outcomes.

Characteristics	Alive or transferred	Lost to follow-up	Died
**Total, no. (%)**	***145 (56*.*4)***	***47 (18*.*3)***	***65 (25*.*3)***
**Age at diagnoses (yr)**			
Median	9.4	9.7	11.2
Range	0.1–29.1	0.1–22.7	0.1–17.3
**Parents’ education level (%)**			
Very low	4 (2.8)	22 (46.8)	15 (23.1)
Low	71 (49.0)	15 (31.9)	43 (66.1)
Acceptable	70 (48.3)	10 (21.3)	7 (10.8)
**Living conditions score (%)**			
Very low	7 (4.8)	15 (31.9)	11 (16.9)
Low	69 (47.6)	25 (53.2)	37 (56.9)
Acceptable	69 (47.6)	7 (14.9)	17 (26.2)
**Income**			
Very low	82 (56.6)	41 (87.2)	49 (75.4)
Low	48 (33.1)	4 (8.5)	9 (13.8)
Acceptable	15 (10.3)	2 (4.3)	7 (10.8)
**Rural no. (%)**	29 (20.0)	36 (76.6)	36 (55.4)
**CKD Stage at 1**^**st**^ **visit (%)**			
I	8 (5.5)	1 (2.1)	0
II	28 (19.3)	7 (14.9)	5 (7.7)
III	53 (36.5)	9 (19.2)	5 (7.7)
IV	25 (17.2)	11 (23.4)	10 (15.4)
V	31 (21.4)	19 (40.4)	45 (69.2)
**Years of diseases (yr)**			
Median	3.51	3.91	1.11
Range	0.07–8.91	0.21–7.80	0.01–7.22
**Time to reach the reference centre (%)**			
< 2 h	77 (53.1)	10 (21.3)	22 (33.9)
2–6 h	63 (43.4)	29 (61.7)	32 (49.2)
> 6 h	5 (3.5)	8 (17.0)	11 (16.9)

The proportion of children with CKD stage IV and V at the first visit (141/257) is possibly the most synthetic clinical expression of the unmet need of timely access to medical care in an LIC environment. The specific impact of the three main socioeconomic indicators on the combined end-point of mortality and lost to follow-up is shown in Table 4, which clearly documents the steep gradients across the stratification levels, as well as a lower discriminatory yield of the "income" variable.

**Table 4:** Socioeconomic factors as related to the cumulative outcome of mortality and lost to follow-up.

Mortality + lost to follow-up	Very low		Low		Acceptable	
	No.	%	No.	%	No.	%
**Living conditions score**	26/33	79	62/131	47	24/93	26
**Income**	90/172	52	13/61	21	9/24	37
**Parents’ educational level**	37/41	90	58/129	45	17/87	19

**Fig 1:**
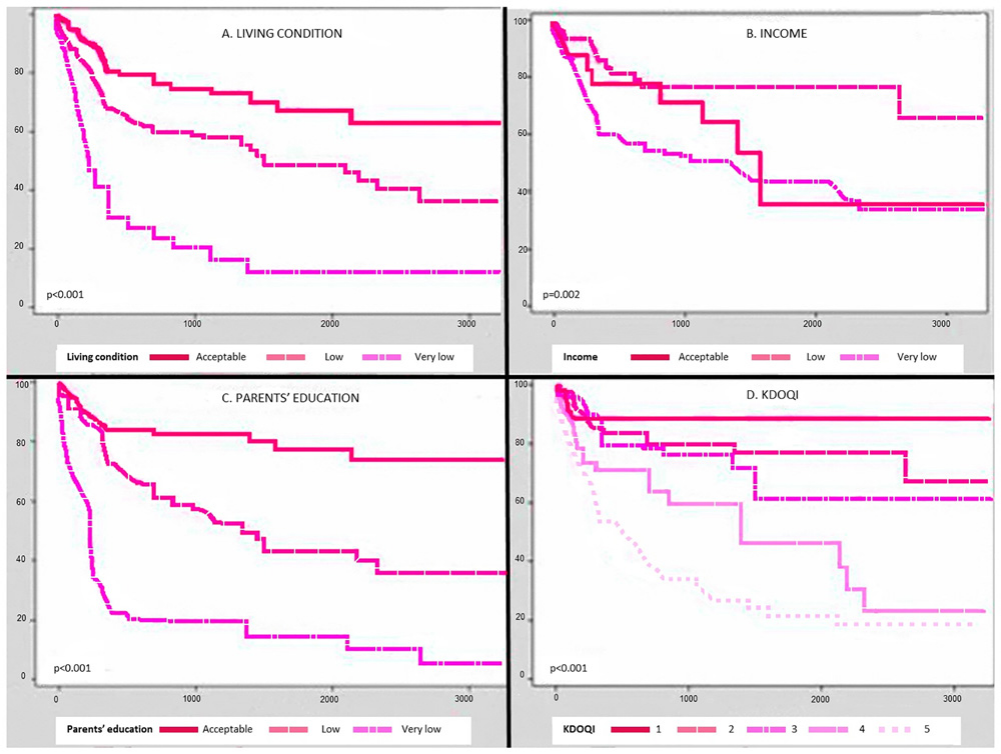
Survival curves. Owing to the great variability of the characteristics of the cohort (from age, to duration of disease, to disease stage at entry, to the different robustness of the socioeconomic markers), only a fully adjusted multivariable analysis of the whole cohort could provide a synthetic view of the determinants of the combined outcome end-point of the cohort as shown in Table 5.

The translation of the above data into survival curves and probabilities is seen in Fig 1, which also allows for the visual comparability of the predictivity of the main clinical variable with the three socioeconomic indicators.

The overlapping HR values obtained for a well-defined clinical marker such as CKD stage, and for those of the apparently most qualitative socioeconomic indicators appear to be the most solid take home message provided by this cohort of children with CKD.

## Discussion

The long-term epidemiological outcome-oriented profile of the cohort of 257 children with CKD presented in this report is, to our knowledge, the first to provide reliable, real-life-based estimates of the influence that socioeconomic and cultural factors have on the hard end-point of mortality, alone and in combination with the equivalent outcome assigned to lost to follow-up.

The descriptive data (Tables 2–4), as well as the results of the Kaplan Meier estimates (Fig 1) and Cox regression analysis (Table 5), are highly consistent in documenting the role of both medical and non-medical risk factors. Table 5, based on the non-censored data covering the whole follow-up period, provides the best comparative summary, as the results have been fully adjusted for the clinical variables at presentation, as well as for the access the cohort patients had to any form of dialysis or transplantation.

**Table 5:** Cox regression analysis.

	Hazard Ratio	CI 95%
Age	0.990	0.941–1.042
**Stage KDOQI**	**2.661**	**1.930–3.668**
**Very low parents‘ education level**	**2.725**	**1.713–4.336**
Very low living conditions score	1.303	0.772–2.197
Very low income score	0.657	0.402–1.074
Rural	0.948	0.456–1.973
Time to reference centre > 6 h	0.979	0.606–1.582

The independent contribution to prognosis, with closely overlapping HR values, of chronic kidney disease stage at diagnosis and of level of education (which is the synthetic expression of the socioeconomic variables), is worth underlining. Advanced clinical stage at diagnosis (up to 55% of our cohort presented at stages IV and V) is, in fact, most likely already the result of the unfavourable effect of chronic exposure to the three non-medical risk factors (where, expectedly, the income level reflects the approximate discriminatory yield of monetary cut-off in a country where informal economy plays a major role), which leads to poor perception of the initial clinical symptoms and, consequently, to extremely late referral to competent nephrological care centers.

On the other hand, unfavourable socioeconomic conditions are definitely contributing to the bad prognosis of the more severe clinical cases, which require strict compliance with frequent medical check-ups, more complex drug treatments and, in the final stage of the disease, the necessity to deal with procedures such as home dialysis and kidney transplantation.

These findings are all the more interesting because the program offered the same "best-care package" to all the children when they were registered in the network database. The progressive expansion of the original hospital-based care facilities meant that assistance was provided to the majority of the country by means of a community-oriented program run by a group of trained health and social workers, who were also trained to ensure the development of an advanced information system for the monitoring and feedback of relevant data.

The results obtained from our cohort are very positive from the point of view of demonstrating the feasibility of a nationwide program for CKD children, as well as their monitoring through an epidemiologically-oriented database. They need, however, to be evaluated and discussed in relation to their outcome endpoints, which appear to be in great excess when compared with the "reference cohorts" used as the basis from which institutional recommendations are formulated[17–19].

The first and main question to be addressed is whether and how far the "reference cohorts" can be assumed to be truly comparable sources of information, specifically in respect to the role played by non-medical determinants of care and outcomes.

Two significant reports have recently focused their attention on the impact of socioeconomic conditions on the outcome of children with CKD: the International Pediatric Peritoneal Dialysis Network[20], and the US and Canada based study on the progression of glomerular filtration rate (GFR)[21]. Income, expressed as gross national product, appears to be a major determinant of mortality in dialysis patients in the first of the studies, together with standardized height, adopted as a marker of global child morbidity across the wide ranges of national values found in the 30 countries included in the study. Self-reported individual incomes, on the other hand, do not correlate with the degree of clinical worsening measured as GFR, while impaired growth was associated with lower income categories[21]. Neither of these studies could, however, be considered as a reasonable comparator for our cohort, due to the differences in the clinical conditions, the more than doubtful comparability of the European countries, and the even more doubtful comparability of the USA and Canada with an LIC like Nicaragua, and due to the restriction of the socioeconomic indicators on income measures only.

In terms of causality of outcomes, the report on dialysis simply confirms what the global burden of disease study has been repeating for 20 years about any disease condition: in less affluent economies a less comprehensive and accessible availability of resources is associated with higher mortality.

The second report[21] confirms that self-reported individual incomes in a relatively homogeneous population cannot be assumed as a reliable marker (even more for surrogate variables such as GFR and hypertension control), while growth, a more comprehensive indicator of deprived social and economic conditions, reaches a statistical significance for the least favourable strata. Against the above findings, our report, which is the first to specifically explore, in a well-defined prospective cohort, the independent contribution of diverse socioeconomic indicators, could provide a reliable answer to the hypothesis which generated the study. Well targeted, representative, setting-specific, routine-nested epidemiological studies are an indispensable tool for exploring the real interactions between medical and non-medical factors, specifically in view of understanding their avoidability[6, 22–24].

## Conclusions and Perspectives

The apparently contradictory gap between the rather successful implementation of a country-wide, freely-accessible care network for children with CKD in a model LIC context and the worse-than-expected outcome of patients lost to follow-up and deceased, must be seen as a challenging result with relevant implications for health care planning and research. The experience of the long-term program in pediatric oncology developed in the same context in Nicaragua brought to light a similar situation[25, 26]: a well-supported medical program is just one of the instruments which, with flexible adjustments, creates an atmosphere of confidence, timely referral and effective intervention.

A very recent Millennium Development Goals report[27] on overall mortality in children under 5 years of age, strongly supports with clear data these somehow obvious findings: the survival advantages of a health (mainly or only) targeted intervention are restricted to the affluent social classes thus widening, not filling, societal equity gaps.

Additionally, along the lines of recent recommendations clearly formulated for adult populations[9] the conclusions of the project are perfectly consistent with the above findings: country specific health care programs must be nested into locally sustainable development goals, closely monitored for their implementation.

The translation-extension of our results is now underway with an agenda which includes:

1. a better integration of chronic pediatric conditions into primary care strategies in order to promote prevention and early timely referral;
2. the consideration of socioeconomic conditions as a mandatory component of the packages of best-care;
3. the formulation and flexible adaptation of guidelines and educational programs based on the information generated by a context-specific epidemiological monitoring of needs and outcomes, guaranteed by an effective database.

It is clear[28] that the increasing pressure of development models based on planned inequality and which interpret universal health coverage in terms of a mix of private and public insurance policies, more than a strategy for making human rights specifically accessible to those who must need them, is not a promising post-2015 scenario.
